# Supplementary material for: A novel assessment method for COVID-19 humoral immunity duration using serial measurements in naturally infected and vaccinated subjects
Source: PLoS One. 2022 Sep 29;17(9):e0274553. doi: 10.1371/journal.pone.0274553 (PMC9521896; doi:10.1371/journal.pone.0274553)
Supplement: S1 Table — Dilution Factors corresponding to 50% reactivity for the first sample is presented for the 6 different antibodies. First and Last sample* indicates the time lapse in days since first symptoms of infection. N = number of samples for each subject. “≤ 6” means that the DF50-value was estimated below limit of quantification. Half-lives are obtained from the linear regression model of ln(DF50) against time in case the model returned a negative slope. Half-life greater than last sample time or when the model returned zero or positive slopes were annotated as > last sample day. Half-lives were indicated as Non-Estimable (NE) when slopes could not be estimated due to low first sample DF50 values (≤50). (DOCX) [file pone.0274553.s001.docx]

**S2 Table. Individual results for the 20 naturally infected subjects.** Dilution Factors corresponding to 50% reactivity for the first sample is presented for the 6 different antibodies. First and Last sample* indicates the time lapse in days since first symptoms of infection. N = number of samples for each subject. “≤ 6” means that the DF50-value was estimated below limit of quantification. Half-lives are obtained from the linear regression model of ln(DF50) against time in case the model returned a negative slope. Half-life greater than last sample time or when the model returned zero or positive slopes were annotated as > last sample day. Half-lives were indicated as Non-Estimable (NE) when slopes could not be estimated due to low first sample DF50 values (≤50).

|  |  |  |  |  |  | | **DF50 of first sample** | | | | | | **Half-life (days)** | | | | | |
| --- | --- | --- | --- | --- | --- | --- | --- | --- | --- | --- | --- | --- | --- | --- | --- | --- | --- | --- |
| **Patient** | **N** | **Age** | **Sex** | **First Sample*** | | **last sample*** | **MP1** | **NP1** | **NP2** | **RBD** | **S1** | **S2** | **MP1** | **NP1** | **NP2** | **RBD** | **S1** | **S2** |
| 1 | 5 | 50 | F | 32 | 267 | | 16 | 680 | 38 | 1102 | 376 | 81 | >267 | 45 | NE | 106 | 119 | >267 |
| 2 | 5 | 48 | M | 16 | 360 | | 110 | 273 | 435 | 706 | 743 | 112 | 91 | 82 | 66 | 196 | 124 | 240 |
| 3 | 5 | 45 | F | 26 | 358 | | 7 | 859 | 27 | 3620 | 1524 | 434 | NE | 77 | NE | 133 | 115 | 218 |
| 4 | 4 | 38 | M | 33 | 265 | | 6 | 294 | 52 | 11170 | 2325 | 323 | NE | 62 | 80 | 103 | 91 | >265 |
| 5 | 2 | 40 | M | 38 | 127 | | ≤6 | 1086 | 56 | 1346 | 433 | 518 | NE | 42 | 32 | 29 | 34 | 31 |
| 6 | 6 | 58 | M | 29 | 270 | | 25 | 7754 | 80 | 13376 | 5576 | 458 | NE | 25 | NE | 43 | 37 | 154 |
| 7 | 2 | 33 | M | 34 | 127 | | 17 | 394 | 255 | 1876 | 489 | 5365 | NE | 16 | 17 | 11 | 15 | 10 |
| 8 | 7 | 27 | F | 26 | 270 | | 7 | 44 | 10 | 229 | 129 | 93 | NE | NE | NE | 117 | 137 | 187 |
| 9 | 4 | 48 | F | 41 | 267 | | 65 | 353 | 25 | 1759 | 549 | 1299 | 75 | >267 | NE | 927 | >267 | 194 |
| 10 | 6 | 55 | M | 37 | 359 | | ≤6 | 88 | 137 | 5781 | 897 | 610 | NE | 74 | 90 | 67 | 129 | 111 |
| 11 | 5 | 52 | M | 38 | 358 | | 10 | 1387 | 53 | 6667 | 3096 | 1203 | NE | 60 | 108 | 120 | 123 | 142 |
| 12 | 4 | 40 | F | 34 | 267 | | 26 | 87 | 7 | 261 | 77 | 284 | 226 | 86 | NE | 170 | 256 | 113 |
| 13 | 5 | 39 | F | 24 | 268 | | ≤6 | 536 | 18 | 566 | 262 | 53 | NE | 45 | NE | >268 | >268 | >268 |
| 14 | 6 | 44 | M | 19 | 270 | | ≤6 | 1794 | 34 | 768 | 607 | 239 | NE | 37 | NE | 87 | 91 | 87 |
| 15 | 5 | 23 | M | 19 | 267 | | 55 | 53 | ≤6 | 255 | 55 | 39 | 169 | 75 | NE | 164 | 230 | NE |
| 16 | 5 | 50 | F | 19 | 267 | | 11 | 234 | 23 | 474 | 216 | 90 | NE | 148 | NE | >267 | 223 | >267 |
| 17 | 6 | 30 | M | 19 | 356 | | 125 | 172 | ≤6 | 2240 | 941 | 213 | 74 | 66 | NE | 126 | 136 | 186 |
| 18 | 6 | 33 | M | 16 | 265 | | ≤6 | 149 | 49 | 366 | 124 | 30 | NE | 60 | 101 | 120 | 153 | NE |
| 19 | 9 | 53 | F | 33 | 357 | | 12 | 324 | ≤6 | 508 | 407 | 51 | NE | 118 | NE | 168 | 130 | >357 |
| 20 | 4 | 60 | M | 23 | 268 | | 16 | 54 | ≤6 | 331 | 230 | 1637 | NE | 155 | NE | 119 | 103 | 76 |
| **Median** | **5** | **44** |  | **29** | **268** | | **11** | **309** | **31** | **935** | **461** | **262** | **-** | **66** | **-** | **120** | **127** | **187** |
