# Supplementary material for: A novel assessment method for COVID-19 humoral immunity duration using serial measurements in naturally infected and vaccinated subjects
Source: PLoS One. 2022 Sep 29;17(9):e0274553. doi: 10.1371/journal.pone.0274553 (PMC9521896; doi:10.1371/journal.pone.0274553)
Supplement: S1 File — (DOCX) [file pone.0274553.s002.docx]

# S1 File: Deriving an optimal dilution sequence

In this supplement, we describe the process that was used for deriving the optimal dilution sequence. The optimal set of dilutions and their corresponding dilution factors were determined a-priori by testing a subset of the serum samples of the naturally infected group. Forty samples (2 samples per patient) were serially diluted and tested. We used the following dilutions: 1/50, 1/100, 1/200, 1/400, 1/800, 1/1600, 1/3200 and 1/6400.

The following criteria were considered:

1. The linear ranges of the dilutions should overlap (for all markers) to ensure accurate quantification.
2. The width of the quantifiable range should be large enough so that observed maximal concentrations are covered.
3. The number of dilutions used is minimized.

Based on the results we sub-selected 3 dilutions to form an optimal sparse sequence. We describe the procedure for deriving the optimal set in detail below.

## Estimated linear ranges

To ensure the first criterion the linear ranges of the individual markers need to be investigated. For this we used the same modelling strategy as described in the statistical methods section of the main text. This model was fitted on the data stemming from the 40 dense dilution sequences. In the model the linear ranges of the markers are parameterised by the hillslope of the markers. The estimated hillslopes coming from the 40 samples are listed in S1 Table 1.

**S1 Table 1. Estimated hillslopes for each marker.**

| **Marker** | **Hillslope** |
| --- | --- |
| MP1 | 1.00^a^ |
| NP1 | 0.73 |
| NP2 | 0.84 |
| RBD | 0.97 |
| S1 | 0.94 |
| S2 | 0.76 |

^a^ Indicates the largest hillslope.

The hillslopes parameterize the width of the linear ranges. A larger hillslope leads to a smaller linear range and vice versa. The hillslope is important for determining the maximum safe distance (first criterion) between two dilutions in the sparse sequence. A higher hillslope with smaller width requires taking smaller steps between selected dilutions. For example, with steps of 3 dilution factors: 1/50 followed by 1/400 versus steps of 4 dilution factors: 1/50 followed by 1/800. Since we aim for overlapping linear ranges, the marker with the highest hillslope determines the maximum step length. This marker is MP1 (S1 Table 1).

From the MP1 curve (S1 Fig 1) we determined that the linear range has at least a width of 4 steps of factor 2 (DF50 = 12.5 – 200). The curve can also be used to determine a safe maximum pixel intensity that lies within the linear range, which is equal to 80.


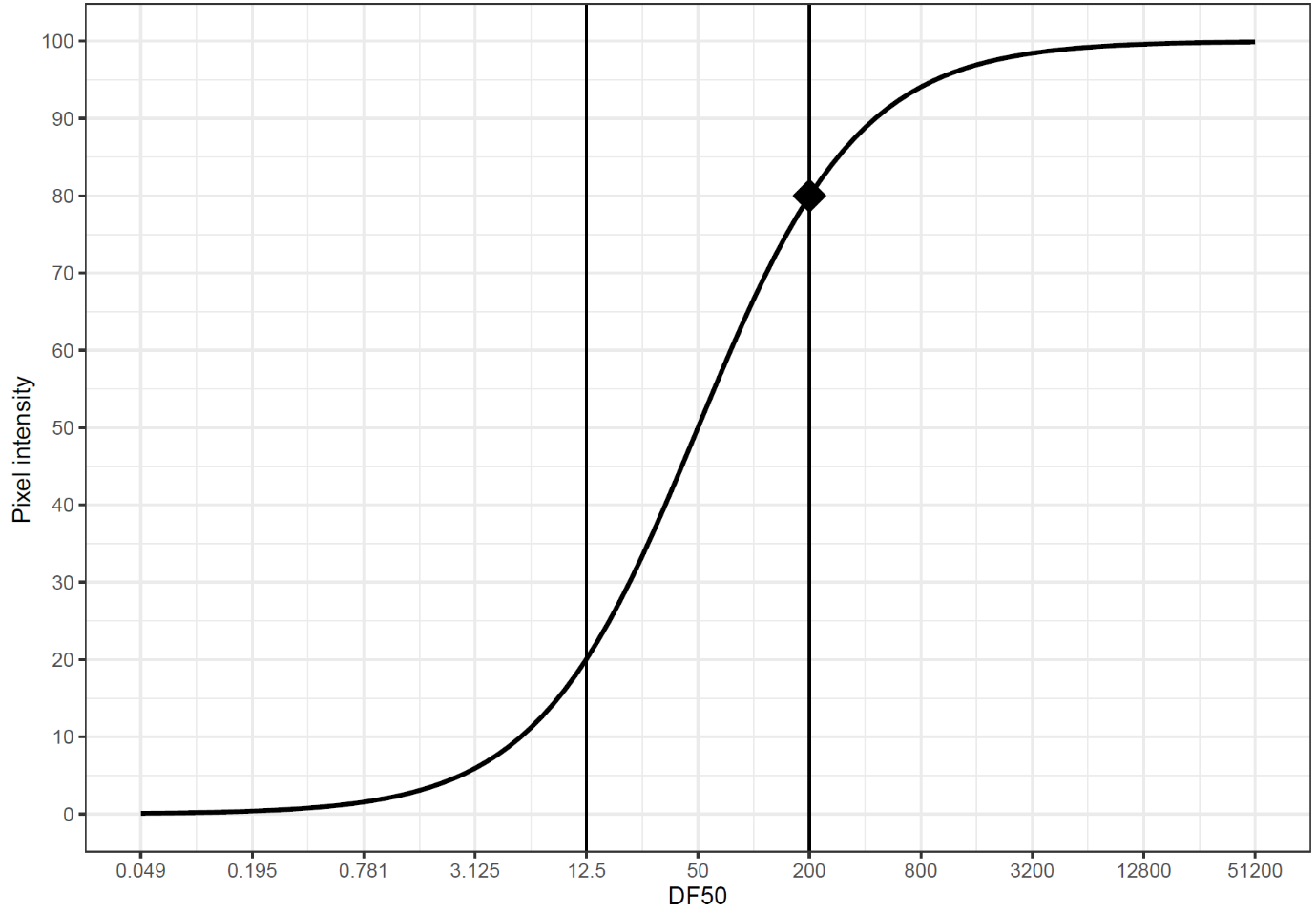
 **S1 Fig 1. Sigmoidal curve of MP1**. This is the sigmoidal curve of MP1 with hillslope = 1.00 and the midpoint of the curve set at DF50=50. The vertical lines indicate the boundaries of the linear range. The diamond indicates the maximum pixel intensity of the linear range (80). Note that the midpoint of the curve is arbitrarily set. A midpoint is necessary to plot the curve, but this does not affect the shape and is therefore not relevant for the analysis.

## Determining the maximum acceptable dilution

Given the maximum pixel intensity in the linear range (pixel intensity=80), we can determine the highest dilution required. The highest dilution should ideally not contain pixel intensities that are beyond the linear ranges of the markers. Pixel intensities below 80 are safe as discussed above. Dilution 1/800 has one value greater than 80, and multiple other values close to this threshold (S1 Fig 2). For dilution 1/1600, all pixel intensities fell well below 80. Therefore, we selected 1/1600 as the highest dilution required. Dilutions 1/3200 and 1/6400 are also safe to use because maximum pixel intensity decreases when sera is more diluted.

##
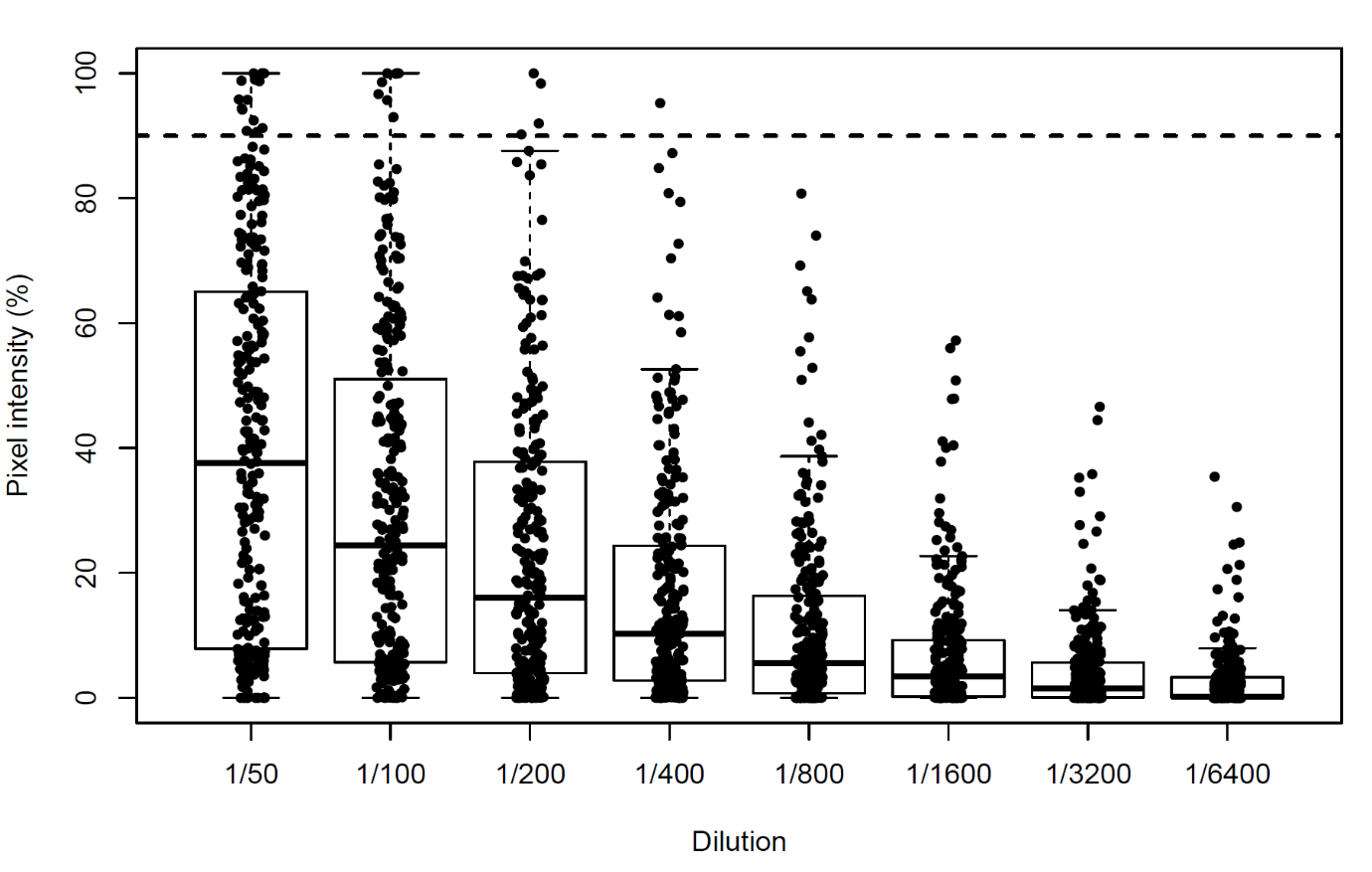


**S1 Fig 2. Pixel intensities per dilution for all markers and samples.**

## Determining the final dilution sequence

Based on the analyses described above, we selected an optimal sparse dilution sequence. First, we fixed the lowest dilution to 1/50. This allows for the reasonable quantification of low antibody concentrations. Second, we choose to take steps of 3 dilution factors. Based on the curve width analysis steps of 4 should be possible. To be safe however, we allowed for some overlap and choose to take steps of 3. Therefore, the second selected dilution was 1/400. Third, since our maximum acceptable dilution must be at least 1/1600 to quantify the maximum observed concentrations, we included an additional dilution. We set this third dilution at 1/3200. We chose 1/3200 instead of 1/1600, because it extends the limit of quantification at no additional cost and takes a step of three dilution factors. We thus selected the set: 1/50, 1/400 and 1/3200 as the optimal set. This sparse set meets our 3 original criteria in that the linear ranges of the dilutions overlap for all markers; the width of the quantifiable range is large enough to cover observed maximal concentrations; and the number of dilutions used is minimized.
