# Supplementary material for: A novel assessment method for COVID-19 humoral immunity duration using serial measurements in naturally infected and vaccinated subjects
Source: PLoS One. 2022 Sep 29;17(9):e0274553. doi: 10.1371/journal.pone.0274553 (PMC9521896; doi:10.1371/journal.pone.0274553)
Supplement: Attachment — Submitted filename: Response to Reviewers.docx [file pone.0274553.s005.docx]

**Editor**

**1. Please ensure that your manuscript meets PLOS ONE's style requirements, including those for file naming.**

We have modified both the title page and the manuscript to comply with the style requirements.

**2. We note that you have stated that you will provide repository information for your data at acceptance. Should your manuscript be accepted for publication, we will hold it until you provide the relevant accession numbers or DOIs necessary to access your data. If you wish to make changes to your Data Availability statement, please describe these changes in your cover letter and we will update your Data Availability statement to reflect the information you provide.**

We have provided an Excel database with the raw value measurements that were used to produce the presented results in our manuscript. This database is now submitted as a supplement (S4 file).

**3. One of the noted authors is a group or consortium “Covid ser study group”. In addition to naming the author group, please list the individual authors and affiliations within this group in the acknowledgments section of your manuscript. Please also indicate clearly a lead author for this group along with a contact email address.**

The individual authors and affiliations within this group are now mentioned in the acknowledgments section of our manuscript. A lead author, together with a contact email address has now been indicated as well.

**4. Please include captions for your Supporting Information files at the end of your manuscript, and update any in-text citations to match accordingly.**

The captions for a supplementary files have been added at the end of our manuscript.

**Reviewer #1**

**The manuscript submitted by Jasper de Boer and colleagues, titled “A novel assessment method for COVID-19 humoral immunity duration using serial measurements in naturally infected and vaccinated subjects” presents a new assessment method for COVID-19 humoral immunity duration which can convert a conventional qualitative assay into a quantitative assay using serial measurements. Although this is a worthwhile study, the manuscript needs a major revision and contains several weak points that authors should address before the manuscript is published. While I understand that English is likely not the authors' first language, the manuscript would greatly benefit from grammatical and verbiage revisions from one more skilled in English. I highlighted a few of the errors below.**

**Minor Comments:**

We thank the reviewer for their extensive list of comments. Most points mentioned below were addressed as suggested, unless mentioned otherwise.

**1. Pages 2, please rephrase the aim clearly in the Abstract, “This study aimed to propose a novel method to assess humoral immunity against SARS-CoV-2 antigens.”**

**2. Page 2, the word “respectively” is placed at the middle of the sentence, it must be at the end.**

**3. Page 3, an APA in-text citation should be placed before the final punctuation mark in a sentence, preferably, please move citation #1 to the end of the sentence.**

We have fixed this recurring issue throughout the manuscript.

**4. Page 3, please delete the sentence: “As there is no antiviral drug to treat the disease at this moment”**

**5. Page 3, please rephrase “ now that massive …” to “With the implementation of large-scale vaccination programs, it is critical to determine the duration of vaccine-induced immunity …"
6. Page 3, please rephrase “At this moment, a consistent depiction of …” to “There is currently no consistent depiction of …. ”**

**7. Page 3, second paragraph, there is a left parenthesis but no corresponding right parenthesis, please fix the error.**

The right parenthesis is placed at the end of the sentence.

**8. References should be presented according to journal guidelines. Referencing is extremely poor across the manuscript. For example, citations 3, 4, and 5 are not cited in the correct format, they should be merged, please revise all in-text citations across the manuscript.**

The in-text citations now comply with the guideline of the journal.

**9. Page 3, the sentence “The duration of immunity is, of course, a very important ..” looks informal, please rephrase.**

The sentence has been rephrased to “The duration of immunity is an important […]”

**10. Page 4, please change the word “collection”, I suggest rephrasing the sentence to “serum samples were collected from two groups: vaccinated and naturally infected subjects”**

**11. Page 4, please add a comma before “out of which”**

**12. Please rephrase “Patients were all symptomatic” to “All of the patients were symptomatic”.**

**13. Please change “collection” to “group”, throughout the manuscript.**

The text has been rewritten to use ‘group’ instead of ‘collection’.

**14. Page 5, in the sentence “before the second injection of vaccine corresponding to 4 weeks after the first dose for participants”, please remove “for participants”.
15. Please be consistent when referring to study subjects, either use “subjects” or “participants”.**

The be consistent we have chosen to replace ‘participants’ with ‘subjects’.

**16. I suggest deleting unnecessary subtitles under the “Methods” section, such as “Ethical Statements” and “Patient and public involvement”**

**17. Please make sure to follow the journal's instructions for preparing the tables, particularly with regard to aspects such as the numbering style, titles, formats, etc.**

**18. Page 10, last paragraph, please add a space after the bracket in “(self-)reported no prior”.**

**19. Page 10, last paragraph, please rephrase “Taking RBD …” to “using RBD …”**

**20. Please be consistent with spacing around dashes and numbers, for example in Table 2, please add a space after the dash in “620 (374 –684)”, also in Table 3 in “32[18– 56]”, “708 [375– 1890]”, “1211 [527– 2910]” , and “108 [57– 208]”.**

**21. Page 14, the first paragraph in the discussion section is very long, please split it into 2-3 paragraphs.**

**22. Page 15, please change “can easily be applied” to “can be easily applied”**

**23. The conclusion can be improved. The conclusion should help the readers understand why your research is significant to them. It should be a synthesis of the main concepts, where you may propose new areas for future research investigations if applicable, rather than a recap of the main points covered or a re-statement of your research issue. Also, some sentences are not properly written in terms of clarity and language.**

The conclusion has been rewritten to highlight the significance of the research and how the method can be applied in future studies. The conclusion now covers and concludes shortly on the main concepts of the manuscript.

**24. An APA in-text citation is placed before the final punctuation mark in a sentence, please carefully revise and fix all in-text citations.**

We have fixed this recurring issue throughout manuscript.

**25. In Figure 1, please change “days since symptoms” to “Time after onset of symptoms (days)”**

The legend name of Figure 1 has been changed as suggested to “Time after onset of symptoms (days)”

**Major comments:**

**1. A major drawback to the manuscript is that the standard of English is significantly sub-par and makes it very difficult to read, obscuring the important message being conveyed. This needs considerable review to make it suitable for publication.**

We have implemented many changes to address this issue. We believe that these changes have improved the text considerably in terms of language. We hope that, by implementing these changes, we have adequately addressed this major drawback raised by the reviewer.

**2. The discussion section lacks a detailed discussion of the results, the tables and figures, deserve further explanation in the Discussion section, please cite the tables and figures where appropriate. The discussion should summarize the main findings, compare the findings with other studies, and discuss the implication of the findings and conclusions. The discussion lacks proper referencing and comparison. There are a lot of statements (particularly in pages 16 and 17), which need to be supported by references to increase credibility of the information provided.**

See the answer to major comment 3.

**3. The discussion extremely lacks coherence and flow of thought. The whole section needs to be divided into shorter paragraphs that flow logically from one idea to the next. The manuscript lacks a clear topic sentence at the beginning of each paragraph that would help improve the flow of the paragraphs. For example, in the last paragraph of page 16, the authors started the paragraph with a very poor and extremely short statement “Our study confirms these last findings.” Also, other paragraphs, such as “Studies of Dan et al. and Wu et al. have provided … with previous SARS- CoV-2 infection” are extremely short and dull. The most important sentence in a paragraph is generally the topic sentence, which should clearly state the subject of the whole paragraph. A paragraph should have the following elements to be as effective as possible: unity, coherence, a topic sentence, and adequate development. All of these aspects overlap. Most importantly, an entire paragraph should be focused on a single topic. It should not finish with another or meander between various concepts.**

The discussion has undergone an extensive revision, keeping into account the points mentioned by both reviewers. We believe that by implementing these changes the flow and coherence of the discussion has been significantly improved. Previously unsupported claims have now been backed up by citing relevant literature. With this we hope to have sufficiently addressed major point 2 and 3 of the reviewer.

**Reviewer #2**

**1. The authors of this manuscript are presenting an immunoassay assessment method to measure multiple SARs-CoV-2 antibodies over a period of time.
Commercial kits that can detect, quantify, and even characterize the neutralizing properties of SARS-CoV-2 antibodies are already available. Some of these kits provide multiplex panels to quantitate multiple SARS-CoV-2 antibodies in a single reaction. Moreover, there are kits designed to detects neutralizing antibodies against different variants of SARS-CoV-2. So, one of the major concerns in this article is how different is their method compared to other available kits/methods. The authors should have compared their method with other commercially available kits to evaluate the sensitivity of their test.**

we would like to highlight that multiplex methods are not suitable for side-by-side comparison. Indeed, the serology profile depends on collective antibody responses to each individual targeted antigen. The aim of the presented approach is to evaluate the changes of serology profiles overtime subsequent to waning of antibodies; this evaluation is made for a specific set of antigens. Therefore, a side-by-side comparison cannot be directly performed.

**Methods:**
**2. Please mention the collection time (period) of first sample following natural infection.**
The collection time of first sample was added to the text: “The collection time of the first sample after onset of symptoms was between 14-41 days (median= 27.5).”

**3. In page 5, it is not enough to rely on pre-vaccination blood to exclude previous infection. This should be coupled with patient history.**

See the answer to point 4.

**4. Also, in page 5, it is mentioned that “commercial assays were performed”. Which assays and used for what and where is the results. It is important to expand on this.**

Depending on the samples’ provider, different commercial assays were used for clinical screening purposes. This included a variety of immunoassays such as Abbott, Diasorin, Wantai, InBios, BioMérieux Vidas, Siemens. We have now specified this in the text. Subjects’ history and clinical information are also included in the database (S4 File).

**5. In page 6, which S antigens were used for the assay. For which variant. Please specify.**

S1 antigen is a recombinant protein expressed in HEK293 Cells based on accession number YP_009724390.1 sequence. S2 antigen is a recombinant protein expressed in Baculovirus-Insect Cells based on accession number YP_009724390.1 sequence.

**6. Also in page 6, it mentioned that a validation test was used to validate the immunoassay. Is this a published data. If so, please cite and if not, please provide more details even as supplementary.**

Validation of the Multi-SARS CoV-2 was performed in an independent laboratory SCIENSANO, Belgium and included the testing of well-characterized positive and negative samples. Each antigen is analyzed for its intrinsic specificity and sensitivity using a set of seropositive (n=540; all >14 days post-PCR determination) and seronegative samples (n=270; including pre-pandemic samples) and optimal concentration for each antigen is determined by Receiver Operating Characteristic curves (ROC).

**7. The same thing applies for dilutions selected at the end of page 7. Either add a reference number of more details to show how exactly you reached to the conclusion to use these 3 or 4 dilutions.**

The process of determining the selection of dilutions was not previously published and is in our opinion too long to include in the main text. Therefore, we have added a supplement that describes this process in detail (S1 File).

**8. In page 8, I think it should be j=6 not 7.**

This was indeed a typo and has been corrected.

**Discussion:**

**9. Authors are listing the results of their study and others without comparison in the first two pages.**

We have rewritten the discussion to improve the flow and coherence. The results are now more clearly compared to those found in literature.

**10. In page 14, please add reference about the link between severity and level of antibodies.**
We have added a reference to a paper from Seow  et al. that links disease severity and (peak) levels of antibodies.

**11. In page 15, it is not reliable to compare NP from vaccinated (with no previous infection) and infected individuals.**

We agree, the objective of this comparison was only to identify individuals that have mistakenly declared a naïve status while they had experienced an asymptomatic infection. We have removed this section from the discussion.

**12. Also in the last paragraph of page 15, “estimated half-life of RBD, S1, S2” which group??**
Since the anti-nucleocapsid protein antibodies are not trigged by the vaccine, this sentence referred to the naturally infected group. We have clarified this in the text.

**13. And half-life reported in this study should be compared to those reported in other studies/ by other methods.**

We have added additional comparisons of our reported half-lives to those found in literature.

**14. In page 16 is an example. And why they are talking about nAbs here. This study did not test for neutralizing activity of the antibodies.**

Neutralization antibody profiles were modeled based on in-vitro neutralization results on a partial set of the studied samples. However, nAbs data are not shown in the present manuscript. We have removed this section from the manuscript.

Finally, we like to thank the reviewers and the editor again for their valuable reviews. We believe that our manuscript has been significantly strengthened by addressing the comments and queries raised. We would be glad to respond to any further questions and comments. We look forward to hearing from you regarding our resubmission.

Yours sincerely,

Dr. Maan Zrein, PhD,

CSO of InfYnity Biomarkers

Email: [mzrein@infynity-biomarkers.com](mailto:mzrein@infynity-biomarkers.com)

Tel: + 33 (0)4 72 76 20 29
