# Supplementary material for: A novel assessment method for COVID-19 humoral immunity duration using serial measurements in naturally infected and vaccinated subjects
Source: PLoS One. 2022 Sep 29;17(9):e0274553. doi: 10.1371/journal.pone.0274553 (PMC9521896; doi:10.1371/journal.pone.0274553)
Supplement: Attachment — Submitted filename: 260822 - rebuttal letter.docx [file pone.0274553.s006.docx]

Lyon, August 30, 2022

Dear Editor,

We hereby submit the second revision of our manuscript titled “***A novel assessment method for COVID-19 humoral immunity duration using serial measurements in naturally-infected and vaccinated subjects***” to be considered for publication in ***PLOS ONE***.

Both an unmarked version and a marked-up copy of our revised manuscript are submitted. The marked-up copy highlights the textual changes made during this revision. Based on the comments of reviewer 2, we made a change to the legend of File S4. This file has also been resubmitted. A new figure (Fig 1) highlighting the subject groups, has been submitted as well.

In the remainder of this letter, we will address the points raised by the second reviewer. The points will be highlighted in boldface and our responses will be written below them in lightface.

**Methods**

1. **Thanks for explaining in detail the characteristics of the groups. I would suggest making a chart for easier tracking.**

We have now included a chart that allows for easy tracking of the subject groups (Fig 1).

**Results**

2. **Please replace antigen to antibodies in the results**

We agree with the reviewer that this is a more natural word choice in this section. We have replaced the word ‘antigen’ with ‘antibody’ in this section.

3. **Table 4, group 3, estimated %relative, please use as percentages as for groups 1 and 2.**

We thank the reviewer for highlighting this overseen inconsistency. As suggested, we have modified the unit for group 3, to match the other groups.

4. **Good that you compared antibody titers using other kits, however, according to S4 table there are no differences in titers among the three dilutions when tested by the commercial kits. Your assay on the other hand is showing a decrease in titer compared to your assay. Is there an explanation for this? Please clarify this in results. Also, please add a sentence or two to describe how different are results of assays (especially ones that target S1/S2) compared to your assay.**

The indicated dilutions (1/50, 1/100, 1/400 and 1/3200) have been performed only for the Multi-SARS-CoV-2 assay. All results obtained from the commercial kits were produced following the instructions and using a (single) working dilution as indicated by the corresponding manufacturer. To maintain the long format of the datasheet, these values are thus repeated and therefore do not show a decrease in titers.

We have now also indicated this in the legend of File S4.

As for the comparability of S1/S2 between commercial assays and the Multi-SARS-CoV-2 immunoassay; unfortunately, such comparison would not be relevant nor informative. The multiplex is optimized to produce immuno-profile results not binary results as is the case for screening assays.

**Discussion**

5. **Add “anti” before “RBD and S-protein titers may be particularly important …”**

We have made this change as suggested.

6. **Please rephrase the following paragraph. It is not clear. “These results correspond well with results previously published in literature. Large differences in antibody titer declines have previously been observed between naturally infected subjects and subjects vaccinated with the Pfizer BioNtech vaccine [24]. Moreover, as discussed above, half-life results from both vaccinated and naturally infected groups generally correspond with the literature.….”**

We have rewritten this paragraph to be clearer and more concise:

*“[…] This faster antibody titer decline in vaccinated groups versus naturally infected groups, corresponds with results previously published in literature. For example, larger declines in antibody titers have previously been observed in subjects vaccinated with the Pfizer BioNtech vaccine versus naturally infected subjects [24].”*

We have also removed the confusing last sentence that restates one of the conclusions of the previous paragraphs.

Finally, we like to thank the reviewers and the editor again for their valuable input. We look forward to hearing from you regarding our resubmission.

Yours sincerely,

Dr. Maan Zrein, PhD,

CSO of InfYnity Biomarkers

Email: [mzrein@infynity-biomarkers.com](mailto:mzrein@infynity-biomarkers.com)

Tel: + 33 (0)4 72 76 20 29
